# A Case Series: Methylene-Blue-Related Toxic Anterior Segment Syndrome

**DOI:** 10.7759/cureus.84448

**Published:** 2025-05-20

**Authors:** Omar Alabbasi, Mubarak W Alahmadi, Mohammed G Alsaedi, Ali Zain Abden M AlShammari

**Affiliations:** 1 Ophthalmology: Anterior Segment, King Salman Medical City, Madinah, SAU; 2 Ophthalmology, Ohud Hospital, Madinah, SAU; 3 College of Medicine, Al-Rayan Colleges, Madinah, SAU

**Keywords:** anterior segment oct, descemet’s stripping automated endothelial keratoplasty, descemet’s stripping endothelial keratoplasty, dsek, intraocular dye toxicity, ocular toxicity, phacoemulsification cataract surgery, tass, toxic anterior segment syndrome

## Abstract

An intracameral injection of methylene blue 1% during cataract surgery induces extreme cytotoxicity, primarily affecting the corneal endothelium and iris epithelium. This case series illustrates the clinical presentation, management, and outcomes of three patients who underwent cataract surgery, with unintended exposure to methylene blue.

All patients demonstrated early-onset corneal edema and anterior segment inflammation shortly after surgery. Two patients developed severe, irreversible endothelial decompensation, leading to persistent corneal edema, decreased visual acuity, and eventual need for Descemet’s stripping endothelial keratoplasty (DSEK). In contrast, the third patient experienced transient corneal edema, which gradually resolved with medical therapy, resulting in significant, though incomplete, visual recovery. No intraocular pressure spikes or retinal toxicity were noted.

In conclusion, methylene blue is highly cytotoxic to intraocular structures, particularly the corneal endothelium and iris epithelium. Accidental administration can cause serious and irreversible visual impairment. Vigilant dye verification protocols, enhanced labeling systems, and multidisciplinary checks are critical in preventing such adverse events. Surgeons must remain cautious and ensure that only approved agents are utilized for anterior capsule staining during cataract surgery.

## Introduction

Phacoemulsification is a common ophthalmic procedure whose success largely depends on proper visualization of the anterior capsule. In cases involving mature or white cataracts, the absence of a red reflex can hinder visualization, increasing the risk of capsulorhexis extension or posterior capsule rupture [1]. To enhance visibility in such cases, capsular dyes are employed, including trypan blue, indocyanine green, sodium fluorescein, rose bengal, and gentian violet [2]. Among these, trypan blue (typically 0.025-0.06%) is widely considered the safest due to its selective staining of the anterior capsule, minimal postoperative inflammation, and low endothelial toxicity [2].

While indocyanine green and fluorescein are also used in ocular procedures, they are generally avoided for anterior capsule staining because of suboptimal staining properties and higher potential for toxicity. In contrast, methylene blue is not approved for intraocular use due to its known cytotoxicity to corneal endothelial cells and iris epithelium. Despite this, methylene blue remains available in ophthalmic operating rooms because it is commonly used for external applications such as scleral or episcleral marking in retinal procedures and eyelid surgery [3]. Unfortunately, its similar appearance and packaging to trypan blue can result in inadvertent intraocular use, leading to toxic anterior segment syndrome (TASS), persistent corneal edema, and irreversible endothelial damage requiring corneal transplantation [4].

This series contains three cases of patients who underwent phacoemulsification for cataract extraction and had inadvertent intraocular injection of methylene blue. The cases help illustrate the complications that come with such accidental intraocular injections of methylene blue.

## Case presentation

Case 1

A 62-year-old, medically free male presented to our clinic complaining of gradual vision loss in the left eye over the past year. On examination, visual acuity was 6/6 in the right eye (OD) and hand motion in the left eye (OS). SLE revealed a clear cornea in both eyes, a deep anterior chamber in both eyes, early cataract changes in the right eye, and a white, hard cataract in the left eye. Fundus examination of the right eye showed a flat retina and a healthy disc and macula. The left eye showed a hazy view with a grossly flat retina.

Management Plan

**Figure 1:**
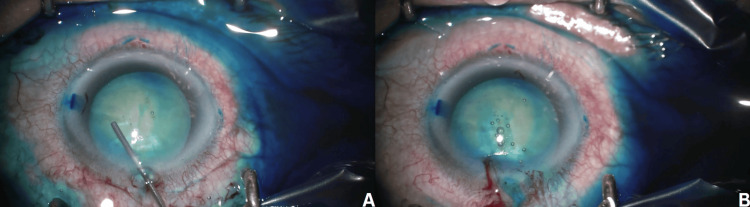
A and B: The conjunctiva was stained by the dye, which appeared darker than the usual dye

We decided to schedule the patient for phacoemulsification with posterior chamber intraocular lens implantation on January 15, 2024. During surgery, while injecting the blue dye into the anterior chamber, the surgeon noticed that the dye appeared darker than usual, as shown in Figure 1A and Figure 1B. The surgeon asked the scrub nurse about the dye, and the nurse confirmed it was trypan blue 0.025%. The surgeon did not verify this himself and proceeded with the surgery without complications. At the end of the surgery, Maxitrol eye ointment was applied, and the eye was patched. The patient was discharged home on moxifloxacin QID and prednisolone acetate 1% Q4H, with instructions to keep the eye patch until the following day.

**Figure 2:**
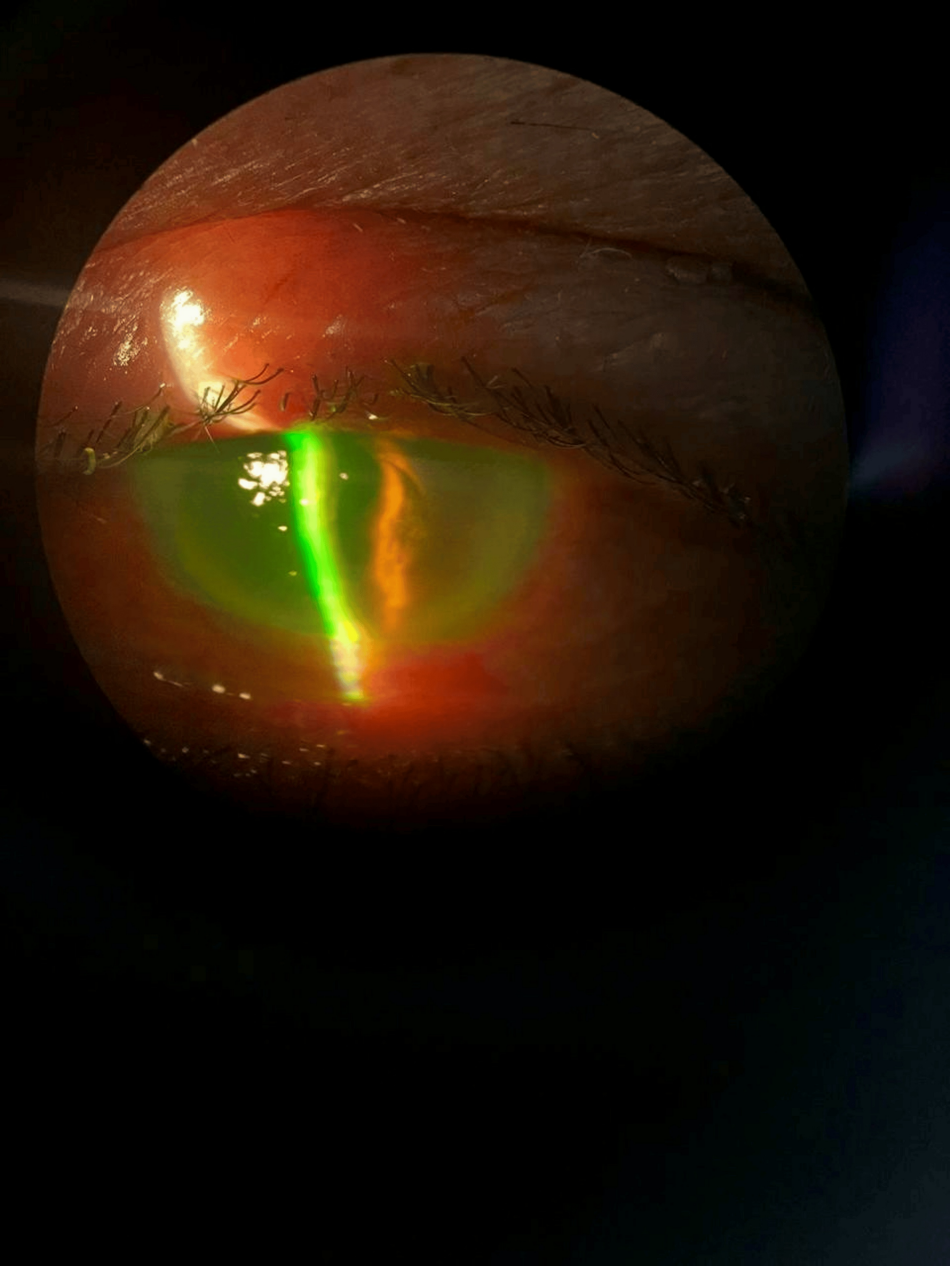
Postoperative day 1 corneal edema

On postoperative day 1, the visual acuity was OS and hand motion. Intraocular pressure was 14 mmHg. Slit-lamp examination (SLE) showed corneal edema (from limbus to limbus), deep anterior chamber, fixed dilated pupil, and intraocular lens in place, with no further view (Figure 2).

The patient was started on prednisolone acetate 1% Q1H, sodium chloride drops QID, and sodium chloride ointment at bedtime. A follow-up appointment was scheduled for one week later. After one week, there was no notable improvement in visual acuity or corneal edema at follow-up. The anterior segment OCT showed no Descemet's detachment. Specular microscopy could not be performed in this case due to persistent corneal edema and media opacity, which precluded accurate endothelial cell count (ECC) measurement.

Upon discussing the case with consultants at the same institution, they reported similar intraoperative findings while using the dye, and postoperative results showed significant toxic anterior segment syndrome. We decided to report this incident to the morbidity department and investigate how this occurred. Upon reviewing the different dyes in the operating room, we discovered that the dye used was not trypan blue 0.025%, but methylene blue. Two weeks later, visual acuity was improved from hand motion to counting fingers. Corneal edema was decreased. The prednisolone acetate dosage was reduced to Q2H, tapering weekly. Sodium chloride drops and ointment were continued. A follow-up appointment was scheduled for one month later.

Six weeks later, the patient's condition remained the same. Prednisolone acetate was discontinued, and sodium chloride drops and ointment were continued. The following three months postoperatively, the patient presented to the emergency room with complaints of pain in the left eye. His visual acuity was counting fingers (CF). SLE showed diffuse corneal edema, plus corneal abrasion, deep anterior chamber, and a round, regular, reactive pupil. The diagnosis of pseudophakic bullous keratopathy was confirmed. The patient was discharged with topical antibiotics and anti-edema eye drops (sodium chloride drops and ointment).

**Figure 3:**
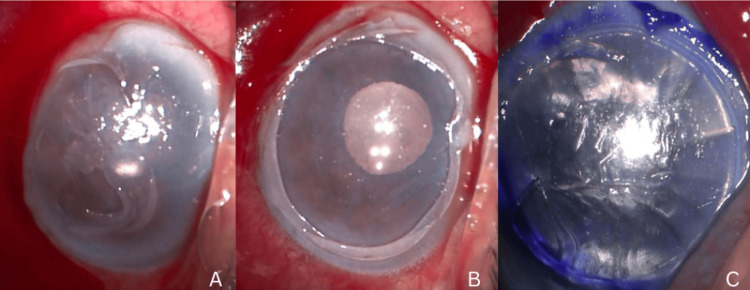
Corneal status A: Preoperative corneal status showing corneal edema; B: After removing the epithelium; C: At the end of the surgery, after DSEK insertion DSEK: Descemet's stripping endothelial keratoplasty

At six months postoperative, the patient was scheduled for Descemet's stripping endothelial keratoplasty (DSEK). Preoperative corneal status was showing corneal edema, before removing the epithelium (Figure 3A), after removing the epithelium (Figure 3B), and after DSEK insertion (Figure 3C).

**Figure 4:**
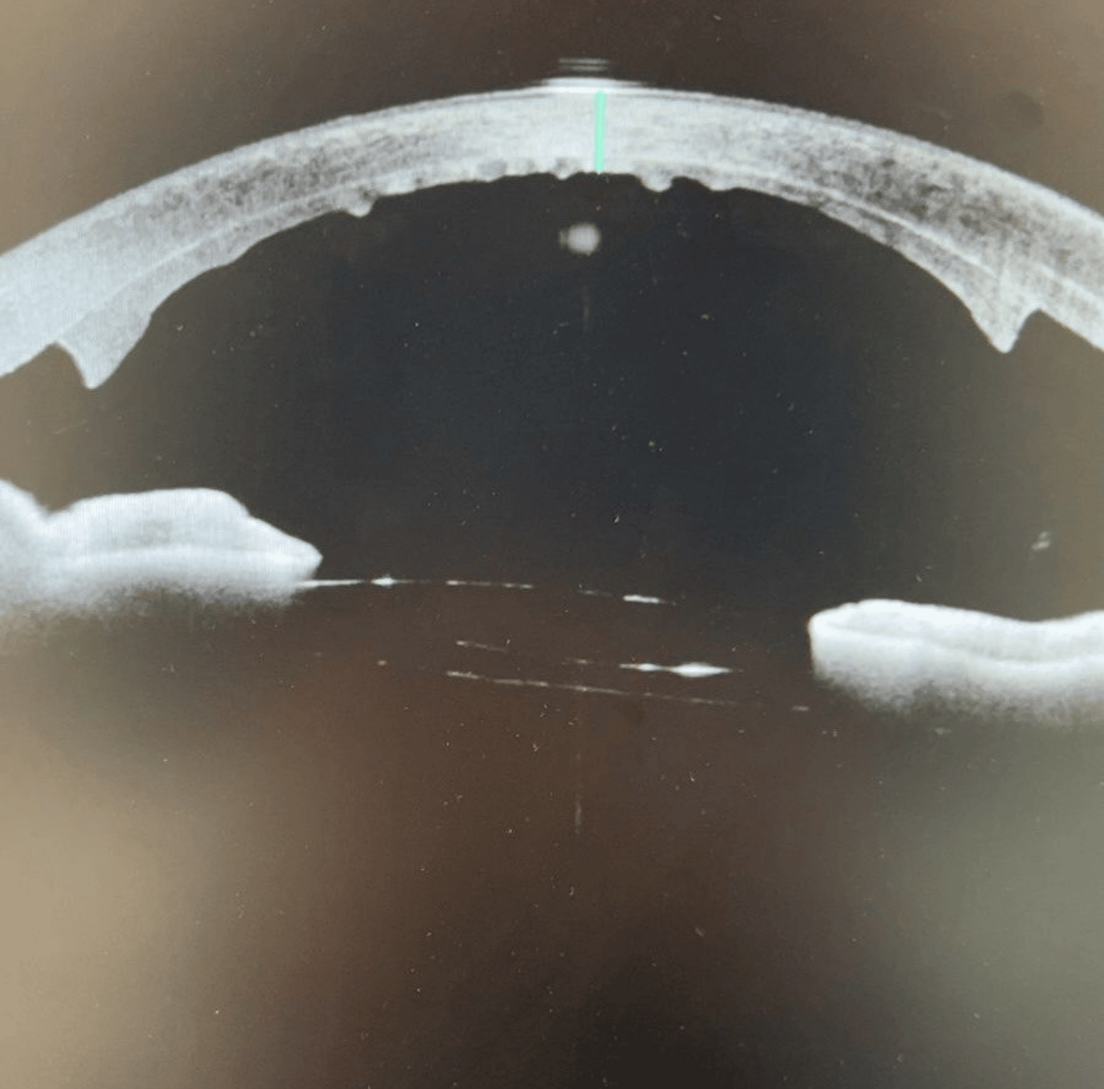
The anterior segment OCT shows an attached lenticule

One day post-DSEK, the IOP was 13. The slit-lamp biomicroscopic examination revealed an attached lenticule with air bubbles (80%), with corneal edema grade 3, and a deep and quiet anterior chamber. Anterior segment OCT was performed, and showed an attached lenticule (Figure 4).

**Figure 5:**
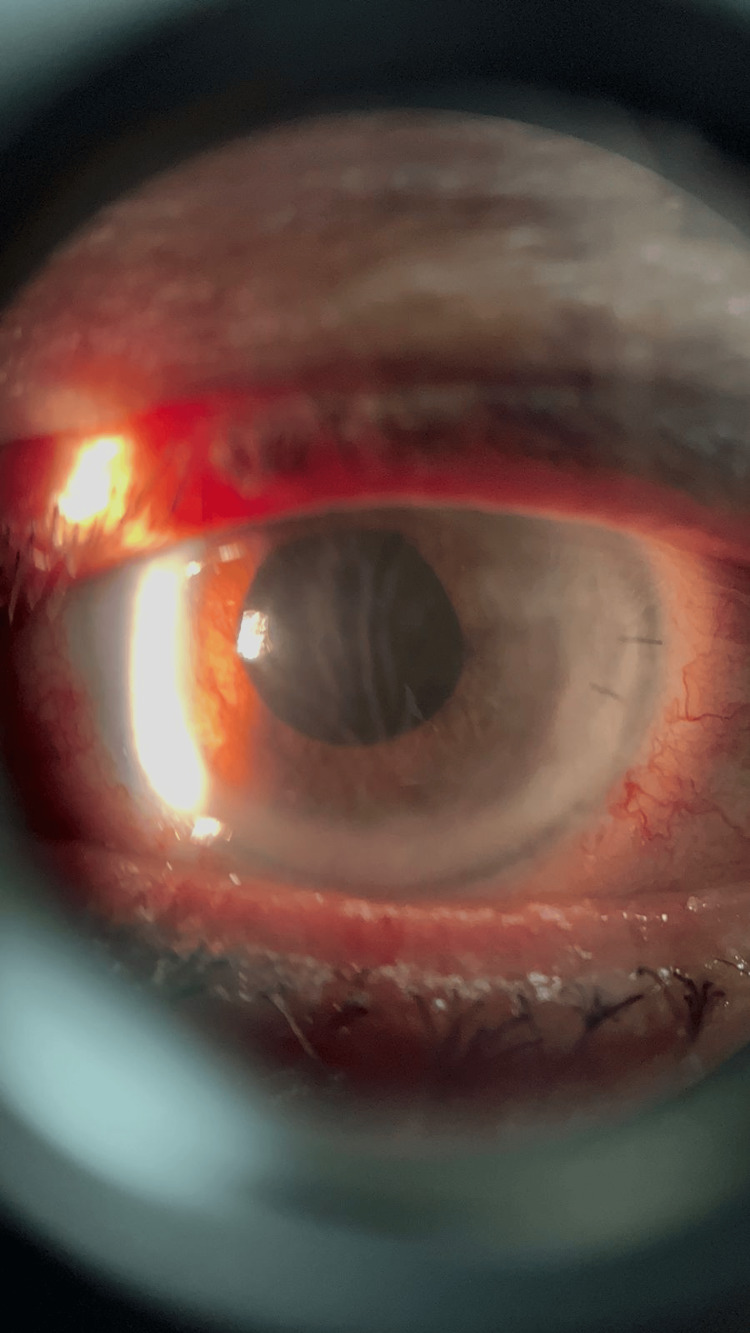
Slit lamp photo shows post DSEK at three weeks DSEK: Descemet's stripping endothelial keratoplasty

Three weeks post-DSEK, the VA was 6/30 while IOP was 15 mmHg. The cornea was stable with a mild Descemet’s fold centrally, with intact sutures as shown in Figure 5, with a deep and quiet anterior chamber.

Case 2

A 60-year-old male from Saudi Arabia presented to the anterior segment clinic with a complaint of gradual vision deterioration in both eyes. Routine ophthalmic examination revealed a visual acuity of 6/36 in the right eye and hand motion in the left eye. Biomicroscopic SLE demonstrated extensive pseudoexfoliation with phacodonesis and a grade 3 nuclear sclerosis cataract in the right eye, as well as a mature cataract in the left eye. Fundus examination of the right eye was unremarkable, with a flat retina and no evidence of retinal tears or breaks. In contrast, the left eye had no view of the fundus, and an ocular ultrasound revealed a normal, flat retina.

Management Plan

**Figure 6:**
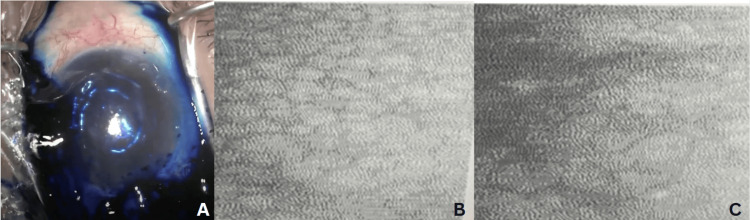
Left eye corneal status and endothelial cell count A: Shows dye appearing darker than usual; B: Endothelial cell count (ECC) six months post-surgery in the right eye; C: ECC six months post-surgery in the left eye; cellular morphology affected in the left eye as compared to the right

The patient was scheduled for left-eye phacoemulsification with posterior chamber intraocular lens (IOL) implantation. In the operating room, it was noticed that the dye appeared darker than the usual dye (Figure 6A). A supplementary video of all three cases can be seen in the Appendices. It shows that the dye is difficult to wash out with balanced salt solution (BSS) cannula or with Healon. Even with phaco probe irrigation, it does not wash out easily. Staining of the iris, the wound, and a blue reflex at the end prior to IOL implantation were also noted.

On day 1 postoperative, the visual acuity was recorded as counting fingers at one meter. SLE revealed severe corneal edema with one superior suture in place, a deep anterior chamber, and the IOL correctly positioned. Treatment included topical antibiotics QID, prednisolone acetate 1% Q1H, sodium chloride drops QID, and sodium chloride ointment at bedtime. Three weeks later, the patient’s visual acuity had improved to 6/18, while intraocular pressure (IOP) was 16 mmHg. Anterior segment examination showed a clear cornea with no edema. Specular microscopy was performed but yielded no readable results. The patient’s visual acuity on the six-month follow-up had further improved to 6/6. SLE was unremarkable, with no significant findings. ECC was performed with significant changes noted on cell morphology, as shown in Table 1 and Figures 6B, 6C. The patient reported a marked improvement in overall symptoms.

**Table 1:** Endothelial cell count (ECC) six months post-surgery; cellular morphology affected in the left eye as compared to the right NUM: Number of Cells Counted; CD: Cell Density; AVG: Average Cell Size; SD: Standard Deviation; CV: Coefficient of Variation; MAX: Maximum Cell Area; MIN: Minimum Cell Area; HEX: Hexagonality %; CT: Central Corneal Thickness; FIX: Fixation

Parameter	Right Eye (R)	Left Eye (L)
NUM	166 (cell)	152 (cell)
CD	2210 (cell/mm²)	2446 (cell/mm²)
AVG	428 (µm²)	366 (µm²)
SD	165 (µm²)	180 (µm²)
CV	38 (%)	49 (%)
MAX	1060 (µm²)	1844 (µm²)
MIN	133 (µm²)	147 (µm²)
HEX	61 (%)	56 (%)
CT	546 (µm)	568 (µm)
FIX	C	C

Case 3

An 81-year-old male with a history of diabetes presented to the ophthalmology clinic, reporting a gradual decline in vision in the right eye. On examination, visual acuity was 6/30 in the right eye and 6/60 in the left eye. IOP was 14 mmHg in the right eye and 12 mmHg in the left eye. SLE revealed a clear cornea with a dense cataract in the right eye. The fundus examination of the right eye was limited by a hazy view, showing a flat retina, while the left eye appeared unremarkable, with no signs of pathology, and was noted to be pseudophakic.

Management Plan

Cataract surgery was planned for the right eye. On post-operation day 1, the patient's visual acuity in the right eye was recorded as CF at 1 meter, and the IOP was 14 mmHg. SLE showed a securely closed surgical wound with no leakage, corneal edema, and a deep anterior chamber. The IOL was correctly positioned, and although a fundus examination was not possible due to poor visibility, a B-scan confirmed a flat retina. To manage postoperative inflammation, the patient was prescribed a tapering regimen of prednisone eye drops, initially every 2 hours, followed by every 3 hours, every 4 hours, every 6 hours, every 8 hours, every 12 hours, and once daily over the course of 1 week. Additionally, sodium chloride 5% was prescribed four times daily for one month.

**Figure 7:**
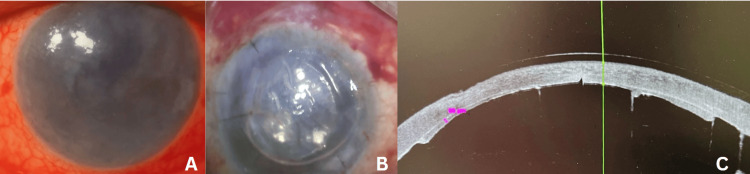
Corneal status A: Persistent corneal edema after three months; Figure B: Day 1 post DSEK; Figure C: As OCT shows attached lenticule (day 1 postoperative) As OCT: anterior segment optical coherence tomography; DSEK: Descemet's stripping endothelial keratoplasty

Two weeks later, the patient's visual acuity in the right eye remained (CF), with IOP measured at 12 mmHg. SLE revealed persistent corneal edema, and the patient continued on the prescribed regimen. At one month postoperative, there was no significant improvement in visual acuity, which remained at CF, and IOP was 10 mmHg. Corneal edema persisted on SLE. ECC measurements were not obtainable in this case due to sustained corneal edema and compromised optical clarity, limiting our ability to assess endothelial cell status objectively. Given the ongoing symptoms and lack of visual recovery, the patient was maintained on sodium chloride 5% and a tapered regimen of prednisone. Due to the persistent corneal edema, DSEK was performed after three months of no improvement. Figure 7A shows persistent corneal edema. Figure 7B shows corneal status after DSEK on Day 1. Visual acuity was hand motion, IOP was 16, and Figure 7C shows the attached DSEK lenticule on anterior segment (AS) OCT.

## Discussion

Methylene blue and trypan blue, both commonly used in the operating room, are generic brands stored in multi-use bottles but have significantly different indications and safety profiles [2]. Trypan blue is widely recognized as the preferred dye for anterior capsule staining in cataract surgery, particularly because it selectively stains the basal membrane of the anterior capsule [2]. On the other hand, methylene blue is used for multiple purposes in the operating theater, including skin marking during eyelid surgery (blepharoplasty) and scleral marking during scleral buckling [3]. In general hospitals, methylene blue is also used by other departments such as urology. However, methylene blue can have different effects, including severe toxicity. Due to its cytotoxic effects, methylene blue is not intended for intraocular use, as it is highly toxic to corneal endothelial cells and the iris epithelium [4]. Nevertheless, the effects of inadvertent intraocular injection of methylene blue have not been extensively investigated.

Methylene blue’s toxicity arises from its ability to generate reactive oxygen species (ROS) and to inhibit mitochondrial complex IV (cytochrome c oxidase). These mechanisms not only disrupt cellular respiration but also induce oxidative stress, resulting in apoptotic death of corneal endothelial cells [5]. Additionally, MB is a relatively small, lipophilic molecule that penetrates intraocular tissues rapidly, leading to widespread toxicity involving both the endothelium and iris epithelium.

In contrast, trypan blue has a larger molecular size and lower membrane permeability, which limits its cytotoxic potential. It stains the anterior lens capsule selectively and is rapidly diluted and removed from the anterior chamber. This pharmacologic profile contributes to its strong safety record in ophthalmic surgery [6].

The cases covered in this series illustrate how the use of methylene blue can lead to significant postoperative complications, including severe corneal edema, fixed dilated pupils, and the need for additional surgical interventions. In the first case, inadvertent use of methylene blue led to the development of bullous keratopathy after persistent corneal edema, ultimately requiring keratoplasty. In the third case, the patient experienced persistent corneal edema and a lack of significant visual recovery. However, in the second case, upon reviewing the surgical video recording, we noticed that methylene blue dye was injected after the viscoelastic material was introduced into the anterior chamber. This may have formed a protective barrier between the corneal endothelium and the dye, potentially explaining the absence of long-term endothelial damage in this case. This patient experienced substantial improvement in corneal edema and visual acuity. Unlike the other two cases, in this instance, the dye was not injected immediately at the side port with direct exposure to the corneal endothelium.

The outcomes noted in these cases are consistent with previous findings that documented the severe anterior segment toxicity associated with methylene blue. For example, in a study by Brouzas et al., exposure to methylene blue caused irreversible corneal damage and persistent iris discoloration [7]. In another study by Timucin et al., it was reported that the corneal damage caused by methylene blue is so significant that although immediate irrigation of the anterior chamber following methylene blue injection mitigated the damage, complete resolution was not always achieved [8]. In the study by Timucin et al., continuous anterior chamber irrigation for 30 minutes led to significant improvements in patient outcomes related to corneal edema and visual acuity, yet subtle stromal haze persisted even after two years [8]. Therefore, early recognition of the inadvertent use of methylene blue and aggressive management may contribute to reducing long-term complications.

The cases also highlight the importance of adequate surgical safety protocols to avoid such errors from happening. The inadvertent use of methylene blue in those cases that happen on the same day illustrates a lack of robust medication verification systems in the operating room. According to Nanji et al., the implementation of standardized labeling protocols, separate storage for high-risk medications, and mandatory two-step verification systems leads to a significant reduction in the risk of intraoperative drug errors [9]. Elsewhere, Samost-Williams et al. showed that standardized verification protocols led to a 40% reduction in errors in perioperative medication administration [10]. Therefore, as evidenced by the adverse effects in our cases and supported by existing literature, ophthalmic surgical teams should adopt stricter dye verification protocols for improved outcomes.

## Conclusions

The use of methylene blue during cataract surgery poses several risks that can lead to severe ocular toxicity. These risks highlight the need for robust medication verification protocols, early intervention strategies, and further research into optimal management approaches. Ophthalmologists working on such cases must be vigilant to avoid errors that could lead to adverse patient outcomes. However, when such errors occur, early identification of toxicity signs and immediate intervention measures, such as anterior chamber irrigation, should be prioritized. Moreover, hospitals should ensure enhanced labeling, improved storage protocols, and comprehensive staff education to prevent such incidents and improve patient outcomes in ophthalmic surgery.
